# App-Based Ecological Momentary Assessment of Problematic Smartphone Use During Examination Weeks in University Students: 6-Week Observational Study

**DOI:** 10.2196/69320

**Published:** 2025-02-05

**Authors:** Ji Seon Ahn, InJi Jeong, Sehwan Park, Jooho Lee, Minjeong Jeon, Sangil Lee, Gangho Do, Dooyoung Jung, Jin Young Park

**Affiliations:** 1 Institute of Behavioral Science in Medicine Yonsei University College of Medicine Seoul Republic of Korea; 2 Department of Psychiatry Yongin Severance Hospital Yonsei University College of Medicine Yongin Republic of Korea; 3 Center for Digital Health Yongin Severance Hospital Yonsei University Health System Yongin Republic of Korea; 4 Graduate School of Health Science and Technology Ulsan National Institute of Science and Technology Ulsan Republic of Korea; 5 Medical Research Team Digital Medic Co., Ltd. Seoul Republic of Korea; 6 Department of Biomedical Engineering Ulsan National Institute of Science and Technology Ulsan Republic of Korea; 7 Digital Medic Co., Ltd. Seoul Republic of Korea

**Keywords:** problematic smartphone use, PSU, ecological momentary assessment, EMA, GPS tracking, digital phenotypes, psychosocial measures, university students, academic stress, mobile health, mHealth, mobile phone

## Abstract

**Background:**

The increasing prevalence of problematic smartphone use (PSU) among university students is raising concerns, particularly as excessive smartphone engagement is linked to negative outcomes such as mental health issues, academic underperformance, and sleep disruption. Despite the severity of PSU, its association with behaviors such as physical activity, mobility, and sociability has received limited research attention. Ecological momentary assessment (EMA), including passive data collection through digital phenotyping indicators, offers an objective approach to explore these behavioral patterns.

**Objective:**

This study aimed to examine associations between self-reported psychosocial measures; app-based EMA data, including daily behavioral indicators from GPS location tracking; and PSU in university students during the examination period.

**Methods:**

A 6-week observational study involving 243 university students was conducted using app-based EMA on personal smartphones to collect data on daily behaviors and psychosocial factors related to smartphone overuse. PSU was assessed using the Korean Smartphone Addiction Proneness Scale. Data collected from the Big4+ app, including self-reports on mood, sleep, and appetite, as well as passive sensor data (GPS location, acceleration, and steps) were used to evaluate overall health. Logistic regression analysis was conducted to identify factors that significantly influenced smartphone overuse, providing insights into daily behavior and mental health patterns.

**Results:**

In total, 23% (56/243) of the students exhibited PSU. The regression analysis revealed significant positive associations between PSU and several factors, including depression (Patient Health Questionnaire-9; odds ratio [OR] 8.48, 95% CI 1.95-36.87; *P*=.004), social interaction anxiety (Social Interaction Anxiety Scale; OR 4.40, 95% CI 1.59-12.15; *P*=.004), sleep disturbances (General Sleep Disturbance Scale; OR 3.44, 95% CI 1.15-10.30; *P*=.03), and longer sleep duration (OR 3.11, 95% CI 1.14-8.48; *P*=.03). Conversely, a significant negative association was found between PSU and time spent at home (OR 0.35, 95% CI 0.13-0.94; *P*=.04).

**Conclusions:**

This study suggests that negative self-perceptions of mood and sleep, along with patterns of increased mobility identified through GPS data, increase the risk of PSU, particularly during periods of academic stress. Combining psychosocial assessments with EMA data offers valuable insights for managing PSU during high-stress periods, such as *examinations*, and provides new directions for future research.

## Introduction

### Background

Smartphones are integral to modern life [1-3], embedding themselves into daily routines owing to their advanced features [4,5] and seamless connectivity to web-based services [6,7]. Convenience and accessibility make smartphones indispensable for many, leading to habitual use and, in some cases, unchecked overreliance [5]. Therefore, excessive smartphone use has raised concerns over its potential adverse effects [6] on interpersonal relationships [6,8], physical [5,9-11] and mental health [12], and daily functioning [13,14]. Research shows that problematic smartphone use (PSU) is linked to negative outcomes, including psychopathology [15], poor academic performance [16], and sleep disturbances [17]. However, PSU is not currently classified as a distinct disorder in major diagnostic manuals, such as the *Diagnostic and Statistical Manual of Mental Disorders, Fifth Edition*, and *International Classification of Diseases, 11th Revision*, highlighting the need to comprehensively explore its impact and influencing factors [14,18,19].

Ecological momentary assessment (EMA) is a methodological approach to capture people’s moods [20,21] and behaviors in their natural environments [22-26]. Recently, EMA has expanded beyond self-report data to include smartphone sensor data [27]. This is known as passive EMA, which describes EMA systems where sensor-based data are collected without any user interaction [26-28]. GPS location–derived features are examples of passive EMA that allow researchers to explore correlations with mental health, such as the proportion of time spent at home [26,29-31]. Recent research has highlighted a significant relationship between increased time spent at home and the severity of depressive symptoms in individuals with major depressive disorder. For instance, an analysis of data from 164 participants using geolocation technology showed that those who spent more time at home during a 2-week period reported more severe depressive symptoms [32,33]. These findings suggest that time spent at home may be an important indicator of mental health, reinforcing the potential of digital phenotyping. By conducting frequent assessments in real-world settings, continuous monitoring through active and passive EMA reduces potential biases associated with traditional self-report methods, which often rely on single-point recollection and introduce inaccuracies [26,28,34,35].

The prevalence of mental health issues among university students is increasing, particularly during examination periods when stress levels peak [36,37]. Academic stress, characterized by increased pressure to perform, has been linked to various challenges, including heightened risks of depression, anxiety, and sleep disturbances [6,38-40], and is positively associated with internet addiction [41,42]. Research indicates that stress can cause excessive use of internet-based devices, potentially resulting in PSU [43]. Studies have also shown a correlation between stress and addictive behaviors related to smartphone use [36]. Moreover, PSU is highly prevalent among university students, with prevalence rates ranging from approximately 10.4% to 70%, depending on demographic and regional factors [44,45]. However, the relationship between PSU and behavioral patterns, such as physical activity, mobility, and sociability, remains underexplored. Limited research has examined how smartphone overuse affects college students’ functionality during examinations. Thus, predicting mental health outcomes using smartphone use and sensor data presents a valuable research opportunity.

### Objectives

This study aimed to investigate the prevalence of PSU among university students and validate previous findings on its associations with depression, anxiety, and sleep disturbance during examination periods. Furthermore, it integrated smartphone sensing data, including GPS-derived behavioral patterns, with self-reported mental health status to explore the impact of PSU on behavior and mental health as well as potential relationships with app-based EMA data.

## Methods

### Study Design

**Figure 1:**
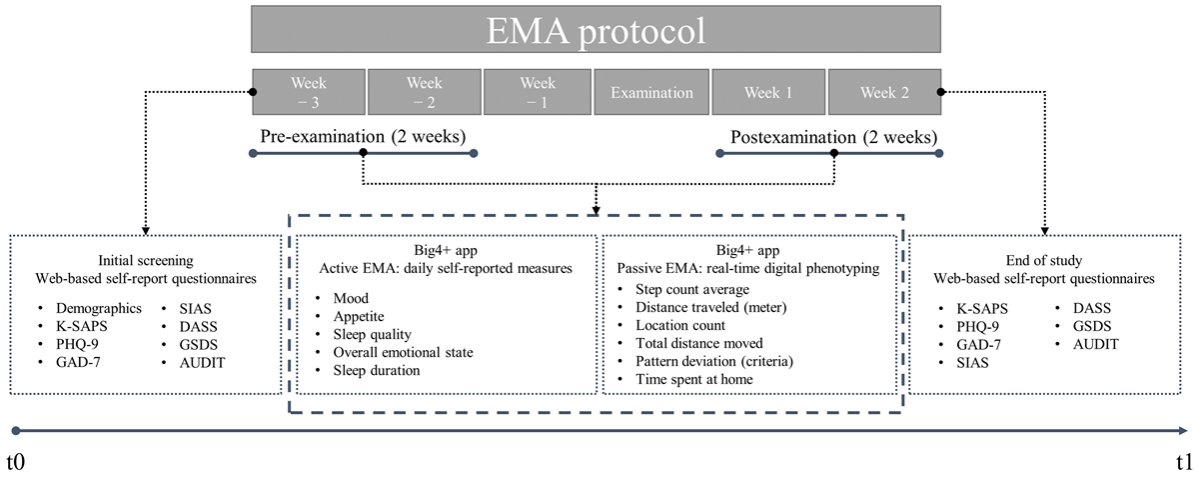
Study design, data collection protocol, and study timeline. AUDIT: Alcohol Use Disorders Identification Test; DASS: Depression Anxiety Stress Scale; EMA: ecological momentary assessment; GAD-7: Generalized Anxiety Disorder-7; GSDS: General Sleep Disturbance Scale; K-SAPS: the Korean Smartphone Addiction Proneness Scale; PHQ-9: Patient Health Questionnaire-9; SIAS: Social Interaction Anxiety Scale.

The study used a prospective observational design to investigate factors related to PSU among university students for 6 weeks. The data were collected during the 2 weeks preceding and following the examinations. Active EMA involved daily self-reported measures, whereas passive EMA captured real-time digital phenotypes. Key psychosocial measures were assessed at baseline and 2 weeks after the examinations (Figure 1). Additional details of the psychosocial assessment tools and EMA measures used in this study are provided in Multimedia Appendix 1.

### Participants

Participants were recruited in 2 waves from the Ulsan National Institute of Science and Technology in Korea during the fall semester, using both web-based and offline posters. Eligibility criteria included being a university student, aged between 19 and 28 years, a native Korean speaker, and owning a smartphone with either Android or iOS. Participants who objected to data collection or had recent suicidal ideation, severe mental illnesses (eg, schizophrenia and intellectual disability), or organic brain disorders were excluded. The study included a web-based survey that collected basic demographic information and responses using validated psychosocial screening tools. All participants provided informed consent, ensuring anonymity. In the first wave, data from 135 participants were collected from September 25 to November 6, 2023, whereas in the second wave, from November 20 to December 31, 2023, data from 108 participants were collected. On completion, participants received a personalized report summarizing their psychosocial assessments and digital phenotype analyzed by a psychiatrist.

### Procedures

#### Web-Based Self-Report Questionnaires

Validated instruments were used to assess the psychological constructs related to smartphone use and mental health. The Korean Smartphone Addiction Proneness Scale (K-SAPS) evaluates excessive smartphone use through 15 items rated on a 4-point scale (ranging from 1 [not at all] to 4 [extremely]), categorizing use as high risk (scores ≥44), at risk (scores 40-43), or normal use (scores <40), with a Cronbach α of 0.814 [46].

The Patient Health Questionnaire-9 (PHQ-9) measured depressive symptoms over the past 2 weeks with 9 statements rated from 0 (not at all) to 3 (nearly every day), yielding a total score between 0 and 27, with higher scores indicating more severe depression [47].

Anxiety levels were assessed using the Generalized Anxiety Disorder-7 (GAD-7), which comprises 7 items scored from 0 to 3, with the total score reflecting the severity of anxiety symptoms [48].

To gauge social interaction anxiety (SIA), the Social Interaction Anxiety Scale (SIAS), comprising 20 items rated from 0 (not at all) to 4 (extremely), was used [49].

The Alcohol Use Disorders Identification Test (AUDIT) was used to screen for alcohol consumption and dependence using 10 statements. Scores >20 for men and >10 for women indicated high-risk drinking, with a Cronbach α of 0.92 [50].

Sleep quality was assessed using the General Sleep Disturbance Scale (GSDS), comprising 21 items on sleep problems experienced over the previous week, rated from 0 (never) to 7 (every day), with total scores ranging from 0 to 147; scores ≥43 indicate significant sleep disturbances [51].

Finally, stress levels were measured with the Depression Anxiety Stress Scales (DASS), which includes 14 items rated on a 4-point scale, ranging from 0 (did not apply to me at all) to 3 (applied to me very much or most of the time), with higher total scores indicating greater stress levels [52].

#### Smartphone Sensing and EMAs

All study participants were required to download and install the EMA digital phenotype data collection app *Big4*+, developed by the Department of Psychiatry, Yongin Severance Hospital, and Digital Medic, on their smartphones before the study began. Throughout the 6-week study period, the Big4+ app continuously collected digital phenotype data, such as distance traveled, step count, and sleep duration, while administering daily EMA surveys that assessed participants’ mental health (mood, appetite, sleep, and overall condition). Participants could monitor the collected data directly through the app. Active EMA measures involved daily responses to 4 mental health–related questions concerning mood, appetite, sleep duration, and sleep quality via the Big4+ app. Each response was collected using a 7-point Likert scale and required approximately 2 minutes to complete. Examples of questions include “How was your mood?” and “Total hours slept last night.” To facilitate engagement, app push notifications reminded users to complete their daily EMA surveys. In parallel, passive EMA data were gathered through the app, which leverages digital phenotyping data from previous research [30]. Data collection commenced once participants provided consent and began using the app, capturing location data every 5 minutes to measure travel distances. This passive data acquisition included metrics such as step count, activity recognition, and sleep data. All the collected data were encrypted and securely stored in a server database. A comprehensive summary of the detailed digital phenotypes is presented in Textbox 1.

**Description of the digital phenotypes.**

**Digital phenotype and description**

- Step count average: the average number of steps taken over a 5-minute interval
- Distance traveled (m): the average distance traveled over a 5-minute interval (m)
- Location count: the number of unique locations visited per day
- Total distance moved: the total distance traveled per day (km)
- Pattern deviation (criteria): the percentage of times a daily routine deviates by >100 m from the established pattern
- Time spent at most frequented location (home): The total time spent (minutes) at the location where the participant spent most of their time during the day

#### Statistical Analysis

To explore the factors related to PSU among university students before and after examinations, the participants with K-SAPS scores ≥40 were classified as the PSU group, while those with scores <40 were classified as the non-PSU group. Age-, gender-, and wave-matched participants without PSU were selected, resulting in a total of 112 participants, including 64 (57.1%) men and 48 (42.9%) women. Independent samples *t* tests (2-tailed) were used for numerical variables, and chi-square tests were used for categorical variables. Continuous variables were reported as mean SD, and categorical variables were reported as n (%).

To identify predictors of PSU, backward multivariable logistic regression analysis was conducted. PSU was the dependent variable, and significant variables in univariate analyses (*P*<.05) were the independent variables. Binary variables were created for each participant to indicate whether their scores on the PHQ-9, GAD-7, SIAS, DASS stress, GSDS, K-SAPS, and AUDIT met or exceeded standardized cutoff values, categorizing participants as “at risk” or “not at risk.” Cutoff values were set at ≥9 for PHQ-9; ≥5 for GAD-7; ≥34 for SIAS; ≥15 for DASS stress; ≥33 for GSDS; and ≥10 and ≥6 for AUDIT for men and women, respectively. In addition, EMA variables were categorized using median split, resulting in “above median” and “below median” groups.

A 2 (group)×2 (time) repeated measures ANOVA was performed to examine the between-group effect (PSU vs non-PSU), within-group effect (pre- and postexamination), and time×group interaction effect. Paired *t* tests compared pre- and postexamination scores within groups, and independent *t* tests assessed group differences at both time points, applying the Bonferroni correction (*P*<.025). All analyses were conducted using SPSS (version 27.0; IBM Corp) for Windows, with a significance of *P*<.05.

### Ethical Considerations

This study is part of a collaborative project between the Department of Psychiatry at Yonsei University and the Department of Biomedical Engineering at Ulsan National Institute of Science and Technology. It was approved by the Ulsan National Institute of Science and Technology Institutional Review Board (UNISTIRB-23-040-A), and it adhered to the principles of the Declaration of Helsinki. All participants were informed about the study’s aims, methods, and data collection procedures and provided their written informed consent before participation. All collected data were anonymized to ensure participant privacy. Participants who completed the 6-week study were compensated with KRW 80,000 (approximately US $60) for their time and effort.

## Results

### Participants’ Demographic Information

A total of 243 students participated in the study, comprising 124 (51%) men and 119 (49%) women aged between 18 and 28 (mean 21.9, SD 1.9) years. The prevalence of PSU was 23% (56/243), with 31 (55%) participants with at-risk level of use (scores ≥40 and <43) and 25 (45%) participants with high-risk level of use (scores ≥43). Out of 243 participants, 56 participants with PSU were identified. To ensure comparability, a matched sample of 56 non-PSU participants was selected based on age, gender, and wave of data collection, resulting in a total matched sample size of 112 participants. This matched sample was used for detailed analyses in the study. Additional demographic characteristics are provided in Table 1.

**Table 1:** Descriptive characteristics of the study population.

			Total participants (N=243)		Study sample (n=112; 1:1 age, gender, and wave matched)^a^
**Sample (N=243), n** **(%)**			243 (100)		112 (46.1)
**Age (y), mean (SD)**			21.9 (1.9)		21.7 (1.8)
**Gender, n (%)**					
	Man	124 (51)		48 (42.9)	
	Woman	119 (49)		64 (57.1)	
**Wave, n (%)**					
	Midterm	135 (55.1)		68 (60.7)	
	Final	108 (44.1)		44 (39.3)	

^a^The study sample consists of 56 participants with problematic smartphone use (PSU) and 56 non-PSU participants matched 1:1 based on age, gender, and wave of data collection. Matching was conducted to ensure comparability between the groups.

### Comparison Between the PSU and Non-PSU Groups at Baseline

The analysis revealed significant differences between the PSU and non-PSU groups in several measures: K-SAPS scores (mean 23.70, SD 5.18 vs mean 44.25, SD 4.22; *P*<.001), PHQ-9 (mean 3.59, SD 4.37 vs mean 6.39, SD 5.51; *P*=.004), GAD-7 (mean 2.18, SD 3.07 vs mean 5.05, SD 5.03; *P*<.001), SIAS (mean 23.21, SD 71.15 vs mean 37.16, SD 17.06; *P*<.001), DASS stress (mean 5.7, SD 8.5 vs mean 11.3, SD 12.2; *P*=.006), GSDS (mean 38.75, SD 17.74 vs mean 48.59, SD 17.27; *P*=.004), and AUDIT (mean 4.91, SD 3.60 vs mean 6.98, SD 4.04; *P*=.005), with the PSU group scoring significantly higher in these assessments. In addition, marginally significant differences were observed in the average distance traveled over a 5-minute interval (mean 157.79, SD 119.24 vs mean 221.87, SD 199.50; *P*=.046). However, daily mood, appetite, sleep, and overall emotional state scores derived from EMA surveys were lower in the PSU group than those in the non-PSU group, although these differences were not significant (Table 2).

**Table 2:** Comparison of means of the characteristics between the PSU^a^ and non-PSU groups at baseline.

Variables			PSU, mean (SD)		Non-PSU, mean (SD)		*t* value (*df*)		*P* value
**Psychosocial measures (PSU, n=56; non-PSU, n=56)**									
	Korean Smartphone Addiction Proneness Scale	44.3 (4.2)		23.7 (5.2)		23.0 (110)		<.001	
	Patient Health Questionnaire-9	6.4 (5.5)		3.6 (4.4)		3.0 (110)		.004	
	Generalized Anxiety Disorder-7	5.1 (5.0)		2.2 (3.1)		3.7 (110)		<.001	
	Social Interaction Anxiety Scale	37.2 (17.1)		23.2 (17.2)		4.3 (110)		<.001	
	Depression Anxiety Stress Scales stress	11.3 (12.2)		5.7 (8.5)		2.8 (110)		.006	
	General Sleep Disturbance Scale	48.6 (17.3)		38.8 (17.7)		3.0 (110)		.004	
	Alcohol Use Disorders Identification Test	7.0 (4.0)		3.6 (4.9)		2.9 (110)		.005	
**Passive EMA^b^** **(digital phenotype; PSU, n=52; non-PSU, n=54)**									
	Step count average	80.7 (47.2)		88.2 (47.3)		–0.8 (106)		.42	
	Distance traveled (m)	221.9 (199.5)		119.2 (157.8)		2.0 (106)		.046	
	Location count	2.7 (0.6)		2.6 (0.7)		0.6 (106)		.54	
	Total distance moved	45.8 (34.3)		57.9 (136.8)		–0.6 (106)		.53	
	Pattern deviation (criteria)	72.3 (24.9)		67.2 (27.0)		1.0 (106)		.32	
	Time spent at home	892.5 (230.9)		935.5 (263.1)		–0.9 (106)		.37	
**Active EMA (PSU, n=52; non-PSU, n=54)**									
	Mood	5.0 (1.1)		5.3 (1.3)		–1.5 (106)		.13	
	Appetite	5.0 (1.1)		5.3 (1.2)		–1.4 (106)		.16	
	Sleep quality	4.7 (1.0)		4.9 (1.2)		–1.0 (106)		.32	
	Overall emotional state	4.9 (1.0)		5.2 (1.2)		–1.3 (106)		.20	
	Sleep duration	430.3 (51.0)		412.9 (61.1)		1.6 (106)		.12	

^a^PSU: problematic smartphone use.

^b^EMA: ecological momentary assessment.

### Factors Associated With PSU in the Univariate Analysis

Univariate analysis revealed significant associations between PSU and multiple psychosocial measures (PHQ-9, GAD-7, SIAS, and DASS stress: all *P*<.01; GSDS: *P*=.02), with no significant difference for AUDIT (*P*=.08). Notably, the PSU group had higher rates of exceeding cutoff scores across the PHQ-9, GAD-7, SIAS, and GSDS. In relation to the digital phenotypes, a significant association was found only for the average distance traveled over a 5-minute interval (*P*=.02). Furthermore, in the active EMA data, only appetite was significantly associated with PSU (Table 3).

**Table 3:** Univariate analysis of the sample characteristics.

Variables					PSU^a^, n (%)		Non-PSU, n (%)		χ^2^ test (*df*)		*P* value
**Psychosocial measures (PSU, n=56; non-PSU, n=56)**											
	**Patient Health Questionnaire-9 (cutoff scores: 9)**								13.2 (1)		<.001
			Not at risk	38 (68)		53 (95)					
			At risk	18 (32)		3 (5)					
	**Generalized Anxiety Disorder-7 (cutoff scores: 5)**								11.7 (1)		.001
			Not at risk	33 (59)		49 (88)					
			At risk	23 (41)		7 (13)					
	**Social Interaction Anxiety Scale (cutoff scores: 34)**								14.8 (1)		<.001
			Not at risk	23 (41)		43 (77)					
			At risk	33 (59)		13 (23)					
	**Depression Anxiety Stress Scales stress (cutoff scores: 15)**								6.9 (1)		.009
			Not at risk	36 (64)		48 (86)					
			At risk	20 (36)		8 (14)					
	**General Sleep Disturbance Scale (cutoff scores: 33)**								5.2 (1)		.02
			Not at risk	11 (20)		22 (39)					
			At risk	45 (80)		34 (61)					
	**Alcohol Use Disorders Identification Test (cutoff scores: male=10, female=6)**								3.0 (1)		.08
			Not at risk	29 (52)		38 (68)					
			At risk	27 (48)		18 (32)					
**Passive EMA^b^** **(digital phenotype; PSU, n=52; non-PSU, n=54)**											
	**Step count average**								0.15 (1)		.70
			Above median	25 (48)		28 (52)					
			Below median	27 (52)		26 (48)					
	**Distance traveled (m)**								5.44 (1)		.02
			Above median	32 (62)		21 (39)					
			Below median	20 (39)		33 (61)					
	**Location count**								0.6 (1)		.44
			Above median	28 (54)		25 (46)					
			Below median	24 (46)		29 (54)					
	**Total distance moved**								1.36 (1)		.24
			Above median	29 (56)		24 (44)					
			Below median	23 (44)		30 (56)					
	**Pattern deviation (criteria)**								1.36 (1)		.24
			Above median	29 (56)		24 (44)					
			Below median	23 (44)		30 (56)					
	**Time spent at home**								1.36 (1)		.24
			Above median	23 (44)		30 (56)					
			Below median	29 (56)		24 (44)					
**Active EMA (PSU, n=52; non-PSU, n=54)**											
	**Mood**								3.8 (1)		.05
			Above median	21 (40)		32 (59)					
			Below median	31 (60)		22 (41)					
	**Appetite**								5.4 (1)		.02
			Above median	21 (40)		34 (63)					
			Below median	31 (60)		20 (37)					
	**Sleep quality**								0.6 (1)		.44
			Above median	24 (46)		29 (54)					
			Below median	28 (54)		25 (46)					
	**Overall emotional state**								3.8 (1)		.05
			Above median	21 (40)		32 (59)					
			Below median	31 (60)		22 (41)					
	**Sleep duration**								1.4 (1)		.24
			Above median	29 (56)		24 (44)					
			Below median	23 (44)		30 (56)					

^a^PSU: problematic smartphone use.

^b^EMA: ecological momentary assessment.

### Independent Predictors of PSU in Multivariable Logistic Regression

Table 4 summarizes the results of the multivariable logistic regression analysis exploring factors associated with PSU based on K-SAPS scores. This analysis included psychosocial measures and digital phenotype data from passive and active EMA, using baseline data collected 3 weeks before the examination. The final model (*R*²=0.424) identified several independent predictors of PSU: PHQ-9 score above the cutoff (B=2.138; odds ratio [OR] 8.480, 95% CI 1.950-36.872), SIAS score above the cutoff (B=1.481; OR 4.398, 95% CI 1.592-12.148), GSDS score above the cutoff (B=1.234; OR 3.436, 95% CI 1.146-10.298), time spent at home below the median (B=–1.066; OR 3.436, 95% CI 1.146-10.298), and sleep duration above the median (B=1.134; OR 3.436, 95% CI 1.146-10.298). The variable “mean distance traveled per 5-minute interval” was not significant (*P*>.05) but was retained in the model. Higher levels of depressive symptoms, SIA, and lower sleep quality, along with less time spent at home, were associated with an increased risk of PSU before the examination.

**Table 4:** Multivariable logistic regression analysis of the effect of the factors on problematic smartphone use.

	B (SE)	Wald	*P* value	OR^a^ (95% CI)
Patient Health Questionnaire-9	2.14 (0.75)	8.13	.004	8.48 (1.95-36.87)
Social Interaction Anxiety Scale	1.48 (0.52)	8.16	.004	4.40 (1.59-12.15)
General Sleep Disturbance Scale	1.23 (0.56)	4.86	.03	3.44 (1.15-10.30)
Mean distance traveled per 5-minute interval	0.82 (0.49)	2.83	.09	2.328 (0.87-5.93)
Time spent at home	–1.07 (0.51)	4.33	.04	0.35 (0.13-0.94)
Sleep duration	1.13 (0.51)	4.90	.03	3.11 (1.14-8.48)
Constant	–2.33 (0.67)	11.91	.001	0.10^b^

^a^OR: odds ratio.

^b^CI not applicable.

### Comparison of Pre- and Postexamination Psychosocial Measures and EMA Data Between PSU and Non-PSU Groups

**Figure 2:**
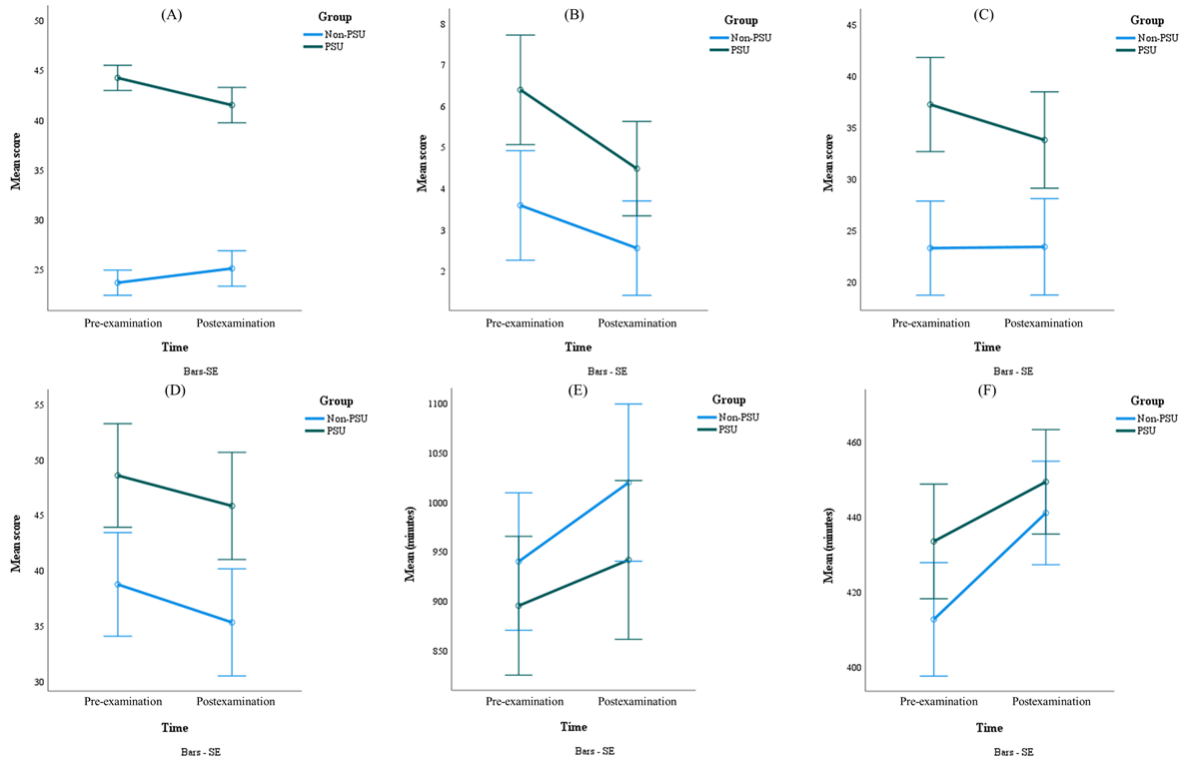
Comparative analysis of pre- and postexamination, psychological and behavioral changes in problematic smartphone use (PSU) and non-PSU groups: (A) PSU (K-SAPS), (B) depression (PHQ-9), (C) social interaction anxiety (SIAS), (D) sleep disturbance (GSDS), (E) time spent at home, and (F) sleep duration. GSDS: General Sleep Disturbance Scale; K-SAPS: the Korean Smartphone Addiction Proneness Scale; PHQ-9: Patient Health Questionnaire-9; SIAS: Social Interaction Anxiety Scale.

Repeated measures ANOVA compared changes in smartphone addiction proneness, depression, SIA, sleep disturbance, time spent at home, and sleep duration between the PSU and non-PSU groups before and after the examination. The results revealed a significant group×time interaction effect for smartphone addiction proneness (*F*_1, 110_=370.29; *P*<.001; ɳp²=0.771), indicating differing patterns of change (Table 5). Specifically, the PSU group exhibited a decrease in average smartphone addiction scores from 44.25 (SD 4.22) to 41.52 (SD 6.72), while the non-PSU group showed an increase from 23.70 (SD 5.18) to 25.13 (SD 6.58). In addition, significant differences were found in depression and SIA. Depression scores in the PSU group decreased from 6.39 (SD 5.51) to 4.48 (SD 5.19), and SIA scores decreased significantly from 37.16 (SD 17.06) to 33.71 (SD 18.52). In contrast, depression scores in the non-PSU group significantly decreased from 3.59 (SD 4.37) to 2.55 (SD 3.13), with no substantial change in SIA scores (Table 6). Furthermore, a repeated measures ANOVA revealed significant changes in sleep disturbance, sleep duration, and time spent at home in both groups before and after the examination. A significant main effect of time for sleep disturbance (*F*_1,110_=5.13; *P*=.02) and a significant group effect (*F*_1,110_=10.94; *P*=.001) were observed. However, the group×time interaction was not significant (*F*_1,110_=.062; *P*=.80; Table 5). Specifically, sleep disturbance or time spent at home did not differ significantly (both *P* values >.05); although sleep duration changed significantly (*P*=.03), it was not significant after applying the Bonferroni correction. In the non-PSU group, while sleep disturbance did not differ significantly (*P*=.07), time spent at home did (*P*=.03), although this was also nonsignificant after correction. However, a significant difference in sleep duration was observed (*P*=.01) (Table 6). Overall, while significant main effects of time were observed for sleep disturbance, time spent at home, and sleep duration (Table 5), paired *t* test results revealed no significant pre- to postexamination differences in sleep disturbance or time spent at home for the groups. However, only the non-PSU group demonstrated a significant increase in sleep duration after the examination, with no change observed in the PSU group (Figure 2; Table 6).

**Table 5:** Group, time, and interaction effects on study variables: repeated measures ANOVA results.

Variables	Group				Time				Group×time interaction		
	*F* test (*df*)	ɳp^2^	*P* value	*F* test (*df*)		ɳp^2^	*P* value	*F* test (*df*)		ɳp^2^	*P* value
K-SAPS^a^	370.29 (1, 110)	0.771	<.001	1.59 (1, 110)		0.014	.21	16.24 (1, 110)		0.129	<.001
PHQ-9^b^	8.55 (1, 110)	0.072	.004	18.98 (1, 110)		0.147	<.001	1.67 (1, 110)		0.015	.20
SIAS^c^	14.79 (1, 110)	0.119	<.001	3.96 (1, 110)		0.035	.049	4.58 (1, 110)		0.04	.04
GSDS^d^	10.94 (1, 110)	0.090	.001	5.13 (1, 110)		0.045	.02	0.06 (1, 110)		0.001	.80
Time spent at home	1.67 (1, 101)	0.016	.20	7.27 (1, 101)		0.067	.008	0.51 (1, 101)		0.005	.48
Sleep duration	2.71 (1, 101)	0.026	.10	17.59 (1, 101)		0.148	<.001	1.40 (1, 101)		0.014	.24

^a^K-SAPS: Korean Smartphone Addiction Proneness Scale.

^b^PHQ-9: Patient Health Questionnaire-9.

^c^SIAS: Social Interaction Anxiety Scale.

^d^GSDS: General Sleep Disturbance Scale.

**Table 6:** Mean differences between pre-examination (baseline) and postexamination evaluations for both groups.

Group and variables			Pre-examination, mean (SD)		Postexamination, mean (SD)		Diff (95% CI)^a^		*t* test (*df*)		Cohen *d*		*P* value^b^
**PSU^c^**													
	K-SAPS^d^	44.25 (4.22)		41.52 (6.72)		2.73 (1.20 to 4.26)		3.58 (55)		0.479		.001	
	PHQ-9^e^	6.39 (5.51)		4.48 (5.19)		1.91 (0.81 to 3.01)		3.48 (55)		0.466		.001	
	SIAS^f^	37.16 (17.06)		33.71 (18.52)		3.45 (0.87 to 6.02)		2.68 (55)		0.358		.01	
	GSDS^g^	48.59 (17.27)		45.84 (18.33)		2.75 (–1.22 to 6.72)		1.39 (55)		0.185		.17	
	Time spent at home	892.52 (230.93)		941.86 (262.94)		–46.22 (–108.81 to 16.38)		–1.48 (50)		–0.208		.14	
	Sleep duration	430.29 (51.03)		449.40 (54.45)		–15.85 (–30.30 to –1.40)		–2.20 (50)		–0.308		.03	
**Non-PSU**													
	K-SAPS	23.7 (5.18)		25.13 (6.58)		–1.43 (–2.82 to –0.03)		–2.05 (55)		–0.274		.045	
	PHQ-9	3.59 (4.37)		2.55 (3.13)		1.04 (0.24 to 1.82)		2.62 (55)		0.35		.01	
	SIAS	23.21 (17.15)		23.34 (16.42)		–0.13 (–2.26 to 2.01)		–0.12 (55)		–0.016		.91	
	GSDS	38.75 (17.74)		35.32 (17.88)		3.43 (–0.33 to 7.18)		1.83 (55)		0.245		.07	
	Time spent at home	935.51 (263.07)		1019.91 (307.08)		–79.63 (–149.21 to –10.04)		–2.30 (51)		–0.319		.03	
	Sleep duration	412.89 (61.13)		441.11 (44.43)		–28.32 (–43.73 to –12.90)		–3.69 (51)		–0.511		.001	

^a^Difference represents the difference between the pre-examination and postexamination mean values (pre-examination – postexamination).

^b^Significance set at *P*<.025 (after Bonferroni correction).

^c^PSU: problematic smartphone use.

^d^K-SAPS: Korean Smartphone Addiction Proneness Scale.

^e^PHQ-9: Patient Health Questionnaire-9.

^f^SIAS: Social Interaction Anxiety Scale.

^g^GSDS: General Sleep Disturbance Scale.

## Discussion

### Principal Findings

This study identified significant associations between PSU and both questionnaire-based demographic and psychosocial measures as well as daily EMA that were either actively or passively collected from a large sample of university students in South Korea. Independent predictors of PSU from psychosocial assessments at baseline included depression, SIA, and sleep disturbance. Conversely, data collected using app-based EMA from baseline to 2 weeks leading up to 1 week before the examination highlighted less time spent at home and longer sleep duration as significant predictors. These findings provide valuable insights into individual daily patterns through real-time, naturalistic measures.

### Comparison With Prior Work

The prevalence of PSU in this study was 23% (56/243), comparable to the 21.7% prevalence reported among Serbian medical students [14] but slightly higher than the prevalence reported among Korean university students [1,53,54]. However, it was lower than the 39.7% prevalence reported by Liu et al [6] regarding smartphone addiction in China. The prevalence of smartphone addiction may vary due to several factors, including differences in measurement scales (eg, cutoff values), sociocultural background, educational environment, sample size, distribution, and technological advancements. Rapid technological progression has likely led individuals to become increasingly accustomed to smartphone use, potentially contributing to the higher prevalence observed in recent research.

Comparison of psychosocial characteristics revealed that the PSU group exhibited significantly higher levels of negative emotions, including depression, anxiety, and stress, as well as more severe sleep disturbances than the non-PSU group. These results suggest a correlation between higher PSU and increased psychological distress and disrupted sleep, consistent with prior findings linking PSU to emotional dysregulation and reduced sleep quality [8,17,55-57]. A significant association was found between PSU and clinical risks for adverse mental health, with a larger proportion of the PSU group classified as being “at risk” for depression, anxiety, stress, and general sleep disturbances compared to the non-PSU group. This suggests a greater likelihood for individuals with PSU to meet clinical thresholds for psychological distress. These findings align with those of a study involving Turkish college students [17], which similarly reported significant associations between smartphone use and elevated depression, anxiety, and poor sleep quality. In addition, recent studies provide evidence of consistent links between smartphone use and psychological factors such as stress, anxiety, and depression [58-61]. Our study reinforces these findings, demonstrating significant associations between PSU and increased risks of depression, anxiety, stress, and sleep disturbances.

Analyzing app-based daily active and passive EMA, this study found distinct GPS-derived movement patterns in individuals with PSU compared to those without it. The digital phenotype data of the PSU group revealed distinct movement patterns, including greater average distances traveled over 5-minute intervals, spending less time at home, and more frequent deviations from their habitual routes, with some movements exceeding 100 m. These patterns, including deviations >100 m from regular paths, may suggest behavioral tendencies such as impulsivity, emotional avoidance, or a heightened pursuit of immediate rewards. Similarly, although the PSU group traveled shorter total distances compared to the non-PSU group (PSU: mean 45.8, SD 34.3 km vs non-PSU: mean 57.9, SD 136.8 km), participants traveled significantly greater distances within 5-minute intervals (PSU: mean 221.9, SD 199.5 m vs non-PSU: mean 119.2, SD 157.8 m). This suggests that while the PSU group covered less ground overall, their movement patterns, characterized by traveling further in shorter periods, may reflect higher impulsivity. The contrast highlights the complex relationship between PSU and impulsive behavior, suggesting that those with PSU may exhibit more erratic movement patterns. These findings align with those of previous studies that link impulsivity to smartphone overuse and related movement behaviors. For example, Pérez de Albéniz Garrote et al [62] found that individuals with high impulsivity often seek novelty and have increased smartphone engagement. Schulz van Endert and Mohr [63] suggested that GPS-tracked movement data can offer objective insights into impulsivity-related smartphone use. Although these studies did not focus explicitly on GPS data, they support our observation [62,63].

The study identified independent significant predictors of PSU in college students before examinations, including psychosocial factors such as elevated levels of depression, SIA, and sleep disturbances. The digital phenotype features extracted from passive EMA revealed that a longer average “distance traveled” (mean distance covered per 5-min interval, measured in meters) and less “time spent at home” (total daily duration at the primary location, measured in minutes) were distinguishing characteristics for students with PSU. In addition, longer sleep duration was an independent predictor in the PSU group. Together, these findings suggest that students with PSU exhibit distinct mobility and sleep patterns before examinations, potentially indicative of broader behavioral tendencies associated with smartphone overuse. Studies on impulsivity and movement patterns [64,65] have shown that individuals with high impulsivity tend to exhibit irregular movement patterns and greater range in travel, supporting the notion that impulsive behaviors may be tied to broader, real-world activity patterns. This could reflect their use of erratic behaviors as a coping mechanism for stress or emotional avoidance.

Specifically, after the examination, smartphone addiction proneness, SIA, and depression were significantly reduced in the PSU group, suggesting that pre-examination PSU may serve as an avoidance coping strategy for academic stress. These results indicate that academic stress can exacerbate mental health vulnerabilities, leading to PSU or worsening existing addiction. Deteriorating mental health is one of the primary risks for smartphone addiction, with addictive behaviors to manage stress and alleviate negative emotions. PSU students may use smartphones to reduce stress and improve mood, increasing the risk of addiction. This finding aligns with previous findings on stress-related digital dependence behaviors. For example, Choi [66] suggests that stress influences smartphone addiction through self-regulation. Moreover, this result is consistent with Kardefelt-Winther’s [67] compensatory internet use model, which proposes that smartphones can help individuals disengage from stress-inducing social situations, with the need for such behavior diminishing as academic pressures ease. These findings are consistent with those of previous studies [68], showing that as stress decreases, smartphone dependence for emotional regulation may also decline. In contrast, the non-PSU group showed a significant increase in sleep duration after examinations, reflecting distinct adaptation mechanisms between the 2 groups.

Another key finding of this study is that SIA was a strong independent predictor of PSU in the students. Unlike general anxiety, SIA is particularly linked to maladaptive smartphone behaviors, potentially due to mechanisms unique to social anxiety that intensify under academic pressures. The heightened vulnerability of students with PSU seems to be compensated for by the portability and flexibility of smartphone apps, which allow individuals to supplement deficiencies in real-life social interactions [6]. These results suggest that high levels of SIA may negatively impact the mental well-being and behavior of students, emphasizing the need for targeted interventions that specifically address SIA. Addressing SIA during examination preparation may be particularly beneficial in supporting the mental health and academic success of students. This finding aligns with reports by Beidel and Turner [69] and Wong et al [70], highlighting the impact of social anxiety on behavioral and academic outcomes, further validating the importance of addressing social interaction challenges within educational settings [69-72].

The findings from this study highlight distinct shifts in smartphone addiction proneness and psychosocial factors between the PSU and non-PSU groups, especially around examination periods. This work demonstrates how varying psychosocial vulnerabilities and coping mechanisms shape smartphone use patterns, with those having PSU exhibiting higher susceptibility to stress and a tendency to rely on smartphones for emotional regulation. Our results emphasize the impact of PSU on mental health and behaviors, linking it to changes in SIA and depression as well as to physical mobility and sleep. These findings are consistent with previous research [67], suggesting that individuals may use digital devices as coping mechanisms for emotional stress. Moreover, the effects of PSU reach beyond digital habits, influencing real-life routines, as shown by studies on movement patterns [64,65]. In conclusion, this study supports the need for interventions focused on reducing SIA and fostering healthy coping strategies among students, aiming to improve both their mental well-being and academic outcomes. Future research should examine the complex interaction between digital behaviors and real-world outcomes, paving the way for a comprehensive understanding of the broader impact of PSU.

### Limitations

This study has several limitations. The generalizability of this study is limited, as the sample comprised students from a single 4-year undergraduate university in Ulsan, South Korea. Consequently, these findings should be applied to different geographical areas or cultural contexts with caution. The sample’s unique characteristics, particularly the fact that all students resided in dormitories, are likely to have resulted in restricted movement patterns, with most mobility occurring within the campus or its immediate vicinity. As all students shared the same institutional environment, they were subject to a uniform set of norms and rules that regulate and constrain behavior, including mobility patterns. This homogeneity extends beyond sociodemographic characteristics to encompass behavioral consistency [73,74], particularly during examination periods. During these high-stress academic periods, shared examination schedules, the necessity of efficient time management, and social pressures to engage in study routines further amplified similarities in daily patterns. Consequently, the findings should also be interpreted within the unique context of academic stress in this demographic, as examination-related pressure is known to influence mobility behaviors and psychosocial factors. While this homogeneity provides methodological advantages, such as reduced variability and enhanced internal validity, it limits the study’s applicability to more diverse populations [73]. To address this limitation, future research should incorporate diverse geographic, educational, and cultural samples to strengthen the external validity of these findings. Including participants from various universities, regions, and cultural contexts would allow for a more comprehensive assessment of the broader applicability of the results. Expanding the sample to include nonstudent populations or community-based cohorts would also help determine whether the observed relationships are unique to undergraduate students or generalizable to individuals with more varied daily routines and environmental constraints.

This study used both active and passive EMA to capture real-time data and digital phenotypes, focusing primarily on GPS-based movement patterns. However, it did not include objective smartphone use metrics, such as screen time or app use patterns. Previous research underscores the critical role of passive smartphone sensing in identifying impulsive behaviors and self-regulation deficits, which often underlie excessive smartphone use [55,75-79]. For instance, hallmark features of PSU, such as compulsive checking and loss of control over smartphone use, can be quantified through objective metrics, providing clearer insights into behavioral manifestations [76,77]. Wen et al [76] presented preliminary evidence linking impulsivity traits to mobile device use. Passive measures, including call logs, battery consumption, and screen time, were found to predict various aspects of impulsivity and impulsive behaviors in nonclinical populations. These findings highlight the importance of integrating diverse passive smartphone metrics into PSU research. Despite the growing body of evidence supporting the utility of passive sensing methods, our study encountered technical challenges in fully using these data sources. Although we successfully extracted passive smartphone metrics, such as app use and screen time, discrepancies in data accessibility between Android and iOS devices limited their integration. For instance, detailed app-level metrics, such as background use and screen time, were available on Android devices but not on iOS devices, which only provided generalized metrics, such as screen-on and screen-off duration. This platform-specific limitation prevented the harmonization of these data streams, ultimately leading to their exclusion from the final dataset. Addressing such technical barriers, including enhancing the cross-platform generalizability of mobile sensing models, is crucial for future research [76]. Overcoming these limitations would enable researchers to integrate diverse data streams, reduce reliance on self-reports, and provide a more accurate and nuanced assessment of PSU.

The results of this study identify significant independent predictors of PSU, including depression, SIA, and sleep disturbance, through regression analysis. While these findings suggest robust associations after adjusting for potential confounders, they are correlational and should not be interpreted as causal. The cross-sectional nature of the baseline psychosocial assessments limits our ability to infer temporal or causal relationships. Future studies using longitudinal or experimental designs will be necessary to establish causality and to explore the underlying mechanisms of these associations. While this study revealed significant differences in GPS-derived movement patterns between the PSU and non-PSU groups, interpreting these patterns as primarily driven by impulsivity or emotional avoidance remains speculative. Several alternative explanations warrant consideration. First, these movement patterns may reflect stress-related coping strategies, wherein individuals engage in physical displacement to manage heightened stress or anxiety during examination periods. Previous research has demonstrated that traveling longer distances is associated with lower levels of stress, greater positive affect, and reduced anxiety. On a daily basis, participants traveling longer distances reported lower levels of stress and anxiety and more positive emotions, while higher levels of routine activity were linked to lower rates of depression and loneliness [80,81]. These findings align with the results of our study. In this study, the non-PSU group traveled significantly longer total daily distances compared with the PSU group; reported significantly lower levels of depression, anxiety, and stress; and exhibited significantly lower rates of routine deviations. The PSU group exhibited shorter total daily distances traveled, but when averaged over 5-minute intervals, participants covered greater distances. This pattern indicates that the PSU group tended to travel farther within shorter periods, suggesting that such increased high-density (short-time long-distance) travel or movement may act as a behavioral response to stress, providing temporary relief or distraction. Second, environmental or lifestyle factors may also contribute to these patterns. Variations in daily schedules, modes of transportation, and academic routines could influence mobility behaviors. For example, students commuting by car or bike may exhibit different movement patterns than those traveling on foot. Similarly, evening activities such as part-time jobs or social gatherings could explain deviations from habitual routes [81]. Third, personal traits, such as conscientiousness or agreeableness, may influence these behaviors. Previous studies have shown that highly agreeable individuals tend to engage in more social interactions and visit a greater variety of locations, whereas those with high conscientiousness often follow structured routines and visit fewer places. These traits may interact with PSU, affecting both mobility and smartphone use behaviors [80]. To further investigate the underlying mechanisms, future studies should incorporate additional behavioral and psychological measures, such as momentary assessments of stress, anxiety, and smartphone use during movement episodes. Integrating these data with GPS metrics could help clarify the roles of impulsivity, emotional avoidance, and other psychological factors in driving these patterns. While this study primarily focused on behavioral patterns, it did not directly assess personal traits such as impulsivity or coping mechanisms, which could provide deeper insight into the psychological factors influencing PSU and mobility. Future research should incorporate validated scales for impulsivity and coping styles, alongside EMA, to better understand how students manage stress and emotional challenges through smartphone use.

### Conclusions

Despite these limitations, the contributions of this study to understanding the relationship between smartphone use and mental health during academic examination periods are significant. First, it was identified that a significantly higher proportion of students with PSU have high risk for depression, anxiety, stress, and sleep disturbances. This underscores the heightened vulnerability of students with PSU under examination-related stress. In addition, this study is among the first to combine active and passive EMA over a 2-week, pre-examination period to capture real-time data on students’ mobility and digital phenotypes. This innovative approach enabled a detailed comparison of movement patterns and behaviors between PSU and non-PSU groups, revealing new insights into how PSU may present in daily life, particularly during high-stress periods. Finally, by including both pre- and postexamination data, this study observed fluctuations in behavioral patterns and mental health outcomes over time, offering a dynamic perspective on the interactions between smartphone use and mental health during critical academic events. These findings align with the concerns raised in the background regarding the negative consequences of PSU. Specifically, this study demonstrated that PSU is associated with heightened risks of depression, anxiety, and stress as well as disrupted sleep patterns, which often exacerbate each other during high-stress periods such as examinations, leading to a compounded negative impact on overall well-being. These results support existing evidence linking PSU to emotional dysregulation, reduced sleep quality, and increased psychological distress. Moreover, the significant associations observed between PSU and clinical risks highlight the importance of addressing these vulnerabilities, particularly during high-stress periods such as examinations. By contextualizing PSU as both a symptom and a contributor to psychological distress, this study emphasizes the need for targeted interventions aimed at mitigating the broader impacts of PSU on students’ mental health and academic performance. Together, these findings represent a meaningful step forward in understanding the real-world implications of PSU in students under academic pressure, providing a foundation for future research in this area.

## Appendix

Multimedia Appendix 1 Korean version of psychosocial assessment questionnaires and daily ecological momentary assessment survey.

## Abbreviations

AUDIT: Alcohol Use Disorders Identification Test
DASS: Depression Anxiety Stress Scales
EMA: ecological momentary assessment
GAD-7: Generalized Anxiety Disorder-7
GSDS: General Sleep Disturbance Scale
K-SAPS: Korean Smartphone Addiction Proneness Scale
OR: odds ratio
PHQ-9: Patient Health Questionnaire-9
PSU: problematic smartphone use
SIA: social interaction anxiety
SIAS: Social Interaction Anxiety Scale

## Data Availability

The datasets generated and analyzed during this study are available from the corresponding author on reasonable request.
