# Supplementary material for: App-Based Ecological Momentary Assessment of Problematic Smartphone Use During Examination Weeks in University Students: 6-Week Observational Study
Source: J Med Internet Res. 2025 Feb 5;27:e69320. doi: 10.2196/69320 (PMC11840384; doi:10.2196/69320)

Korean Smartphone Addiction Proneness Scale, K-SAPS

다음 문항은 스마트폰 사용에 관한 질문입니다. 각 문항들을 주의 깊게 읽고 가장 적합한 칸에 표시해 주시기 바랍니다.

		1	2	3	4
		전혀 그렇지 않다			매우 그렇다
1	스마트폰의 지나친 사용으로 학교 성적이나 업무능률이 떨어졌다.				
2	스마트폰을 사용하지 못하면 온 세상을 잃을 것 같은 생각이 든다.				
3	스마트폰을 사용할 때 그만해야지 라고 생각은 하면서도 계속한다.				
4	스마트폰이 없어도 불안하지 않다.				
5	수시로 스마트폰을 사용하다가 지적을 받은 적이 있다.				
6	가족이나 친구들과 함께 있는 것보다 스마트폰을 사용하고 있는 것이 더 즐겁다.				
7	스마트폰 사용시간을 줄이려고 해보았지만 실패한다.				
8	스마트폰을 사용할 수 없게 된다면 견디기 힘들 것이다.				
9	스마트폰을 너무 자주 또는 오래한다고 가족이나 친구들로부터 불평을 들은 적이 있다.				
10	스마트폰 사용에 많은 시간을 보내지 않는다.				
11	스마트폰이 옆에 없으면, 하루 종일 일(또는 공부)이 손에 안 잡힌다.				
12	스마트폰을 사용하느라 지금 하고 있는 일(또는 공부)에 집중이 안 된 적이 있다.				
13	스마트폰 사용에 많은 시간을 보내는 것이 습관화 되었다.				
14	스마트폰이 없으면 안절부절 못하고 초조해진다.				
15	스마트폰 사용이 지금 하고 있는 일(또는 공부)에 방해가 되지 않는다.				

Patient Health Questionnaire-9, PHQ-9

각 문항을 읽고 지난 2 주 동안 자신이 느끼고 행동한 것을 가장 잘 나타낸다고 생각하는 곳에 표시해 주시기 바랍니다.

		0	1	2	3
		없었다	2 일 이상	일주일 이상	거의 매일
1	기분이 가라앉거나 우울하거나 희망이 없다고 느꼈다.				
2	평소 하던 일에 대한 흥미가 없어지거나 즐거움을 느끼지 못했다.				
3	잠들기가 어렵거나 자주 깼다 혹은 너무 많이 잤다.				
4	평소보다 식욕이 줄었다 혹은 평소보다 많이 먹었다.				
5	다른 사람들이 눈치 챌 정도로 평소보다 말과 행동이 느려졌다 혹은 너무 안절부절 못해서 가만히 앉아 있을 수 없었다.				
6	피곤하고 기운이 없었다.				
7	내가 잘못했거나 실패했다는 생각이 들었다 혹은 자식과 가족을 실망시켰다고 생각한다.				
8	학교 공부, 독서, TV 시청과 같은 일상적인 일에도 집중할 수가 없었다.				
9	차라리 죽는 것이 더 낫겠다고 생각했다 혹은 자해할 생각을 했다.				

Generalized Anxiety Disorder-7, GAD-7

각 문항을 읽고 지난 2 주 동안 다음의 문제들로 인해서 얼마나 자주 방해를 받았는지 해당 되는 곳에 표시해 주시기 바랍니다.

		0	1	2	3
		전혀 방해 받지 않았다	며칠 동안 방해 받았다	7 일 이상 방해 받았다	거의 매일 방해 받았다
1	초조하거나 불안하거나 조마조마하게 느낀다.				
2	걱정하는 것을 멈추거나 조절할 수가 없다.				
3	여러 가지 것들에 대해 걱정을 너무 많이 한다.				
4	편하게 있기가 어렵다.				
5	너무 안절부절 못해서 가만히 있기가 힘들다.				
6	쉽게 짜증이 나거나 쉽게 성을 내게 된다.				
7	마치 끔찍한 일이 생길 것처럼 두렵게 느껴진다.				

Social Interaction Anxiety Scale, SIAS

다음의 문항들은 대인관계 상황에 대해 사람들이 어떻게 느끼는지 알아보기 위한 것입니다. 각 문항을 읽고 자신에게 해당하는 점수에 표시해 주시기 바랍니다.

		0	1	2	3	4
		전혀 그렇지 않다				매우 그렇다
1	나보다 높은 지위에 있는 사람(선생님, 상사 등)과 대화할 때 긴장된다.					
2	다른 사람들과 눈을 마주치기가 힘들다.					
3	나 자신이나 내 감정에 대해서 말해야 할 때 긴장된다.					
4	동료들과 편안하게 어울리는 것이 어렵다.					
5	길에서 아는 사람을 만나면 긴장된다.					
6	여러 사람들과 어울리는 것이 불편하다.					
7	다른 사람과 단 둘이 있는 상황에서는 긴장된다.					
8	모임에서 사람들을 만나는 것이 편안하다.					
9	다른 사람들과 이야기하는 것이 어렵다.					
10	이야깃거리를 쉽게 생각해 낸다.					
11	내 나이 또래의 친구들을 사귀기 쉽다.					
12	이상하게 보일까 봐 나 자신을 표현하는 것이 걱정된다.					
13	다른 사람의 주장에 반대하기가 어렵다.					
14	매력적인 이성과 이야기하는 것이 어렵다.					
15	사회적 상황에서 무엇을 말해야 할 지 몰라 걱정하곤 한다.					
16	잘 알지 못하는 사람들과 어울려야 할 때 불편하다.					
17	대화 도중 부적절한 말을 할 것 같다.					
18	여러 사람들과 어울려 이야기할 때 무시당할까 봐 걱정하곤 한다.					
19	여러 사람들과 어울릴 때 긴장한다.					
20	잘 알지 못하는 사람에게 인사를 해야 할지 망설인다.					

Depression Anxiety Stress Scales, DASS

지난 한 주 동안 아래의 문항이 자신에게 얼만큼 해당되었는지 표시해 주시기 바랍니다.

		0	1	2	3
		전혀 해당되지 않음			꽤 많이 또는 거의 대부분 해당됨
1	나는 안정을 취하기 힘들었다.				
2	입이 바싹 마르는 느낌이 들었다.				
3	어떤 것에도 긍정적인 감정을 느낄 수가 없었다.				
4	숨쉬기가 곤란한 적이 있었다. (심하게 호흡이 가쁘거나 가만히 있을 때도 호흡곤란이 있었다.)				
5	무엇인가를 시작하는 것이 어려웠다.				
6	어떤 상황에 과잉 반응을 보이는 경향이 있었다.				
7	몸이 떨리는 것을 느꼈다. (예: 손 떨림)				
8	모든 일에 신경을 너무 많이 쓴다고 느꼈다.				
9	내가 너무 당황해서 웃음거리가 될까 봐 걱정했다.				
10	나는 기대할 것이 아무것도 없다는 생각이 들었다.				
11	자꾸 초조해졌다.				
12	나는 진정하는 것이 어려웠다.				
13	기운이 처지고 우울했다.				
14	내가 하는 일에 방해가 되는 것을 용납할 수 없었다.				
15	내 자신이 심한 불안상태까지 도달했음을 느꼈다.				
16	어떤 것에도 몰두 할 수가 없었다.				
17	나는 사람으로서 가치가 없다고 느꼈다.				
18	내가 꽤 신경질적이라고 느꼈다.				
19	가만히 있을 때에도 심장이 두근거리는 것이 느껴졌다. (예:심장이 심하게 빨리 뛰는 느낌, 불규칙한 심장 박동)				
20	아무 이유 없이 무서움을 느꼈다.				
21	산다는 것이 의미가 없다는 생각이 들었다.				

General Sleep Disturbance Scale, GSDS

지난 일주일 동안 며칠이나 아래 문항과 같은 상황을 경험하였는지 응답해 주시기 바랍니다.

		0	1	2	3	4	5	6	7
		없음							매일
1	잠들기가 힘들었다.								
2	자다가 중간에 깼다.								
3	너무 일찍 잠이 깼다.								
4	자고 나면 개운했다.								
5	잠을 푹 자지 못했다.								
6	낮에 졸렸다.								
7	낮에 깨어 있으려고 애썼다.								
8	낮에 짜증이 났다.								
9	낮에 지쳐있거나 피곤했다.								
10	수면 상태에 만족한다.								
11	낮에 정신이 맑고 활기찼다.								
12	너무 많이 잤다.								
13	너무 적게 잤다								
14	정해진 시간에 낮잠을 잤다.								
15	예정하지 않은 시간에 잠이 들었다.								
16	잠들기 위해 술을 마셨다.								
17	잠들기 위해 담배를 피웠다.								
18	잠들기 위해 허브 제품을 사용했다.								
19	잠들기 위해 수면제를 사서 먹었다.								
20	잠들기 위해 처방 받은 수면제를 먹었다.								
21	잠들기 위해 진통제를 먹었다.								

Alcohol Use Disorders Identification Test, AUDIT

각 문항을 읽고 자신에게 해당하는 점수에 표시해 주시기 바랍니다.

1	술을 얼마나 자주 마십니까?	전혀 마시지 않음	월 1 회 또는 미만	월 2~4 회	주 2~3 회	주 4 회 이상
2	평소 술을 마시는 날 몇 잔 정도나 마십니까?	0~2 잔	3~4 잔	5~6 잔	7~9 잔	10 잔 이상
3	한번 술을 마실 때 소주 1 병 또는 맥주 4 병 이상의 음주는 얼마나 자주 하십니까?	전혀 없음	월 1 회 미만	월 1 회	주 1 회	매일 또는 거의매일
4	지난 1 년간 술을 한번 마시기 시작하면 멈출 수 없다는 것을 안 때가 얼마나 자주 있었습니까?	전혀 없음	월 1 회 미만	월 1 회	주 1 회	매일 또는 거의매일
5	지난 1 년간 당신은 평소 같으면 할 수 있었던 일을 음주 때문에 실패한 적이 얼마나 자주 있었습니까?	전혀 없음	월 1 회 미만	월 1 회	주 1 회	매일 또는 거의매일
6	지난 1 년간 술을 마신 다음날 아침에 일 나가기 위해 다시 해장술을 필요했던 적이 얼마나 자주 있었습니까?	전혀 없음	월 1 회 미만	월 1 회	주 1 회	매일 또는 거의매일
7	지난 1 년간 음주 후에 죄책감이 들거나 후회를 한 적이 얼마나 자주 있었습니까?	전혀 없음	월 1 회 미만	월 1 회	주 1 회	매일 또는 거의매일
8	지난 1 년간 음주 때문에 전날 밤에 있었던 일이 기억나지 않았던 적이 얼마나 자주 있었습니까?	전혀 없음	월 1 회 미만	월 1 회	주 1 회	매일 또는 거의매일
9	음주로 인해 자신이나 다른 사람이 다친 적이 있었습니까?	전혀 없음	지난 1 년간에는 없었음 (2 점)		지난 1 년간 있었음 (4 점)	
10	친척이나 친구, 또는 의사가 당신이 술 마시는 것을 걱정하거나 술 끊기를 권유한 적이 있습니까?	전혀 없음	지난 1 년간에는 없었음 (2 점)		지난 1 년간 있었음 (4 점)	

Daily EMA survey (Active EMA measures via the Big4+ app)

오늘의 기본검사를 진행합니다.

		1	2	3	4	5	6	7
		매우 나빴다						매우 좋았다
Mood	지난 24 시간 동안 기분은 어떠셨습니까?							
Appetite	지난 24 시간 동안 식욕은 어떠셨습니까?							
Sleep quality	지난 24 시간 동안 잠은 어떠셨습니까?							
Overall emotional state	지난 24 시간 동안 전반적으로 어떠셨습니까?							

Sleep duration	어제 수면시간을 표시해주세요	pm	1	2	3	4	5	6	7	8	9	10	11	12
		am	1	2	3	4	5	6	7	8	9	10	11	12

Mood

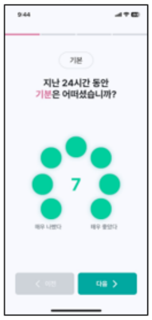

Appetite

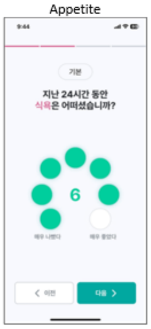

Sleep quality

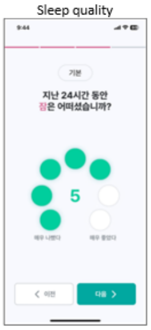

Overall emotional state

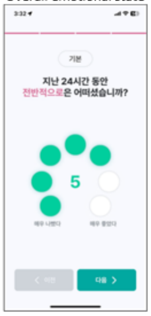

Sleep duration

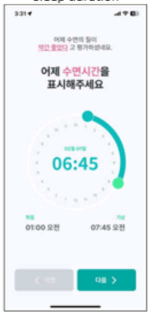

Supplement: Multimedia Appendix 1 [file jmir_v27i1e69320_app1.pdf]
